# New Therapeutics to Treat Castrate-Resistant Prostate Cancer

**DOI:** 10.1155/2013/379641

**Published:** 2013-05-27

**Authors:** Ömer Acar, Tarık Esen, Nathan A. Lack

**Affiliations:** ^1^VKF American Hospital, Guzelbahce sokak, Nisantasi, Istanbul 34365, Turkey; ^2^School of Medicine, Koç University, Rumelifeneri Yolu, Sariyer, Istanbul 34450, Turkey

## Abstract

The effective treatment of castrate-resistant prostate cancer (CRPC) has proven to be very challenging. Until recently, docetaxel was the only therapeutic demonstrated to extend overall patient survival. Yet recently, a considerable number of new therapeutics have been approved to treat CRPC patients. These remarkable advances now give new tools for the therapeutic management of late-stage prostate cancer. In this review, we will examine mechanistic and clinical data of several newly approved therapeutics including the chemotherapeutic cabazitaxel, antiandrogen enzalutamide, endocrine disruptor abiraterone acetate, immunotherapy sipuleucel-T, and bone-targeting radiopharmaceutical alpharadin. In addition, we will examine other promising therapeutics that are currently in Phase III trials.

## 1. Introduction

Prostate cancer is a common disease that affects approximately 1 out 7 men in their lifetime. Fortunately, for most patients, prostate cancer is typically a localized indolent disease that can be effectively managed by either radiotherapy or surgery. However, it is far more difficult to treat those patients with aggressive or metastatic forms of the disease. The current standard of care for those patients who have failed surgery or radiotherapy is typically androgen deprivation therapy (ADT) by either surgical castration or LHRH agonists/antagonists. In prostatic neoplastic cells, androgen deprivation induces cellular apoptosis leading to a reduction in the tumor burden [1]. While being initially effective, the response from ADT is typically temporary and the cancer almost always recurs. As the cancer can proliferate despite castrate levels of androgen, it is defined as a castration-resistant prostate cancer (CRPC). Patients with CRPC have traditionally had very few treatment options available and were primarily given palliative care. In 2004, a classic study by Tannock et al. demonstrated that docetaxel could slow the disease progression and extend CRPC patient survival [2]. While being a considerable step forward, the benefit of docetaxel is relatively limited and can only increase the median survival time by approximately 2-3 months. 

Due to this unmet clinical need, there has been a tremendous effort by both academic and industrial researchers to develop new therapeutics that can slow the progression of both pre- and post-docetaxel CRPC. This has proven to be very challenging, with numerous trials failing to demonstrate improvement over docetaxel [3–5]. However, in the last several years there has been a groundswell of new therapeutics to treat post- and pre-docetaxel patients. For the first time, there are now treatment options available for those patients who have failed docetaxel. While none of these treatments are curative, these additional tools offer the potential to greatly slow the progression of disease and extend patient survival. 

In this paper, we will review the clinical results of several newly approved drugs that extend patient survival including cabazitaxel, enzalutamide, abiraterone, sipuleucel-T, and alpharadin (Table 1). 

## 2. Cabazitaxel

Closely related to docetaxel, this semisynthetic taxane was specifically designed to have low affinity to the multidrug transporter p-glycoprotein 1 [11]. This compound binds to and stabilizes tubulin, thereby causing inhibition of microtubule depolymerization, which results in cell cycle arrest and tumor cell apoptosis. Cabazitaxel was found to have high activity in cell lines with resistance to doxorubicin, vincristine, vinblastine, paclitaxel, or docetaxel [12]. Further, cabazitaxel demonstrated *in vivo* antitumor efficacy on most docetaxel-sensitive, -refractory, or -resistant models, such as B16/TXT melanoma [13]. 

In a Phase I clinical trial, cabazitaxel was found to have an acceptable safety profile with neutropenia as the main dose-limiting toxicity [14]. Other toxicities were generally mild to moderate and included nausea, vomiting, diarrhea, neurotoxicity, and fatigue. Importantly, in this early study a partial response was observed in two patients with late-stage metastatic prostate cancer, including one who had previously been treated with docetaxel. However, it is important to note that it is not known if cabazitaxel's poor affinity for P-glycoprotein is the cause of efficacy in docetaxel-resistant patients [15]. Based on the results from both Phase I trial and also an additional Phase II study in metastatic breast cancer [16], cabazitaxel development was accelerated. In the TROPIC (NCT417079) Phase III trial, a total of 755 CRPC patients, who had progressed after or during docetaxel-based chemotherapy, were randomized to receive either cabazitaxel or mitoxantrone with both arms receiving oral prednisone [6]. Overall survival was the primary endpoint, and progression-free survival (PFS), treatment response, and safety were secondary endpoints. Cabazitaxel was found to extend median OS time (15.1 months) compared with those patients who received mitoxantrone (12.7 months) (hazard ratio (HR) = 0.70, *P* < 0.0001). The cabazitaxel treatment arm also showed significant improvement in PFS (2.8 versus 1.4 months, *P* < 0.0001), objective response rate according to RECIST criteria (14.4% versus 4.4%, *P* < 0.005), and PSA response rate (39.2% versus 17.8%, *P* < 0.0002). The most common grade 3 or higher adverse events included neutropenia (cabazitaxel 82% versus mitoxantrone 58%) and diarrhea (cabazitaxel 82% versus mitoxantrone 58%). Due to the safety profile of cabazitaxel, the administration of the drug requires careful monitoring, dose modification, and potential prophylactic treatment with granulocyte stimulating factor (G-CSF) in high-risk patients. Based on these results, cabazitaxel was approved by the FDA in 2010 to treat docetaxel-refractory-patients with CRPC. 

There are numerous clinical trials currently ongoing to explore various aspects of cabazitaxel utility. This includes trials to directly compare the effects of docetaxel and prednisone vs. cabazitaxel and prednisone (NCT1308567), lower doses of cabazitaxel (PROSELICA; NCT1308580), earlier intervention with cabazitaxel (NCT1718353) and optimization of neutropenia management (PROSPECTA; NCT01649635). 

## 3. Endocrine Disruptors

While being counterintuitive, there is considerable evidence that for most CRPC patients androgen receptor (AR) signaling is still required for tumor growth. This can be clearly observed with the simple fact that during CRPC progression there is a continued increase in the expression of the AR-dependent protein PSA. Indeed, high levels of nuclear AR have been observed in over 80% of patients with CRPC [17, 18]. The results from preclinical experiments with AR targeting therapeutics have clearly demonstrated that “castrate-resistant” cancers are not independent of AR transcriptional signaling [19, 20]. Patients who failed ADT develop resistance through several different proposed mechanisms, including overexpression of either AR [21] or coactivators [22] which sensitize the AR to lower physiological levels of androgen; point mutations which can cause the AR to be promiscuously activated by relatively abundant nonandrogenic steroids; activation/sensitization of the AR through phosphorylation of the protein [23]; and interestingly intracellular *de novo *production of androgen by the tumor itself [24]. Whatever the cause, as AR transcriptional activation is critical to the growth of the cancer, this nuclear receptor offers an effective pharmacological target to treat CRPC patients. 

### 3.1. Enzalutamide

Enzalutamide is an orally bioavailable antagonist that directly acts on the androgen receptor. Originally identified by the iterative optimization of nonsteroidal agonists, enzalutamide was found to have exquisite selectivity for AR over other nuclear receptors [19]. *In vitro *studies demonstrated that enzalutamide could effectively inhibit AR transcriptional activation in bicalutamide-resistant cells [19]. As a more potent derivative of previous antiandrogens, enzalutamide has also been demonstrated to reduce both AR translocation and interaction with coactivators [19]. Interestingly, in current preliminary studies, enzalutamide has yet to demonstrate agonist activity in resistant cell lines, something that was readily observed with previous antiandrogens [19]. However, given the mechanism of action, it is expected that new mutations will be identified from clinical studies that confer agonist activity to enzalutamide. 

In a Phase I/II trial containing 140 CRPC patients, enzalutamide demonstrated encouraging antitumor activity with 56% of these late-stage patients showing a decrease in serum PSA of 50% or more [25]. The most common grade 3-4 adverse event was fatigue (11% of patients), though this was generally resolved at lower doses. These results led to the larger AFFIRM Phase III trial that tested the effect of enzalutamide on OS in docetaxel-refractory patients (NCT974311). In this study, 1,199 men who had received ≤2 regimens of docetaxel-based chemotherapy were randomized to receive either enzalutamide or placebo. Enzalutamide demonstrated a dramatic benefit in patient OS (median 18.4 months versus 13.6 months, HR 0.63; *P* < 0.001) [8, 26]. Furthermore, the drug was found to be superior over placebo in secondary endpoints including serum PSA decline of >50% (54 versus 2%, *P* < 0.001), soft-tissue response rate (29% versus 4%, *P* < 0.001), time to PSA progression (8.3 versus 3.0 months, *P* < 0.001), radiographic progression-free survival (8.3 versus 2.9 months, *P* < 0.001), and the time to the first skeletal-related event (SRE) (16.7 versus 13.3 months, *P* < 0.001). Mild adverse effects were observed including fatigue, diarrhea, musculoskeletal pain, and hot flashes. Interestingly, the incidence of grade 3-4 adverse effects was lower in the treatment arm, suggesting that the majority of these adverse events were actually disease related. Despite concerns from preclinical [27] and preliminary clinical studies [25], the prevalence of seizure was very low (0.6%) in the enzalutamide treatment arm. Based on these promising clinical results, the FDA approved enzalutamide in 2012 for the treatment of post-docetaxel CRPC patients. 

Given the effect of enzalutamide in late-stage CRPC patients, there is considerable interest in Phase III PREVAIL clinical trial that tests the effect of the drug in pre-docetaxel setting (NCT1212991). Recruitment of 1680 patients has been completed, and it is expected that the results will be available in 2014. 

In addition to enzalutamide, the antiandrogen ARN-509 is also currently in clinical development. While this compound has similar *in vitro *potency to enzalutamide, it was demonstrated to be more efficacious per unit dose in mouse models [28]. Given the very similar chemical scaffolds, it will be interesting to see if this compound has activity in enzalutamide-resistant patients. 

### 3.2. Abiraterone

All gonadal steroids, including testosterone and dihydrotestosterone, are produced by the conversion of cholesterol to pregnenolone, which is subsequently converted to androgen. In this metabolic pathway, the enzyme cytochrome P450 17 (CYP17) catalyzes two essential reactions: the hydroxylation of pregnenolone and progesterone at the C_17_ position to generate 17*α*-hydroxypregnenolone and 17*α*-hydroxyprogesterone and also the cleavage of the C_17_–C_20_ bond of 17*α*-hydroxypregnenolone and 17*α*-hydroxyprogesterone to form dehydroepiandrosterone and androstenedione. As these enzymatic steps are critical, inhibition of CYP17 will prevent the synthesis of androgens, while also leading to an accumulation the metabolic precursors mineralocorticoids [29]. As CYP17 represents a critical catalytic step in androgen biosynthesis, this essential enzyme offers a particularly attractive target to treat prostate cancer patients. Inadvertently, CYP17 had previously been targeted with the second-line therapeutic ketoconazole. Originally developed as a broad-spectrum antifungal agent, it was extensively used in the treatment of prostate cancer after it was found to induce gynecomastia due to inhibition of testicular and adrenal androgen synthesis. Despite its proven efficacy, ketoconazole is not without considerable limitations including such side effects as hepatotoxicity, gastrointestinal toxicity, and adrenal insufficiency. Moreover, ketoconazole has considerable cross-reactivity and also inhibits the drug metabolizing enzymes CYP3A4 and CYP24A1. 

Abiraterone was developed to overcome the limitations of ketoconazole. This steroidal CYP17 inhibitor is both more selective against CYP17 and >10 times more potent than ketoconazole [30]. In preclinical studies, abiraterone effectively reduced serum testosterone to below the level of detection [31]. Due to poor bioavailability of the parent molecule, the prodrug abiraterone acetate was used for further clinical development [32]. 

In a Phase I clinical trial, abiraterone acetate was well tolerated and found to decrease tumor burden in both ketoconazole pretreated and naïve CRPC patients [33]. The most common adverse events were fatigue, hypertension, headache, nausea, and diarrhea. Due to the mechanism of action, many of these adverse events were believed to be due to excessive mineralocorticoids and in subsequent trials exogenous corticosteroids such as dexamethasone or prednisone were given with abiraterone acetate. Despite drug-induced hypokalemia, abiraterone acetate had no effect on QT interval [34]. In a Phase II study of 47 docetaxel pre-treated CRPC patients, abiraterone acetate and prednisone was found to be effective at lowering serum PSA level >50% in 51% of patients. The addition of oral prednisone decreased the incidence of hyperaldosteronism-related symptoms including hypokalemia, hypertension, and fluid retention. Based on these promising results, abiraterone acetate was assessed in a large multicenter Phase III trial with docetaxel-refractory CRPC patients [7]. The COU-AA-301 (NCT638690) trial enrolled 1,195 patients who were randomized 2 : 1 to either receive abiraterone acetate plus prednisone or placebo plus prednisone. In the study design, the primary endpoint was OS, while secondary endpoints included time to PSA progression, progression-free survival rate according to radiological findings, and PSA response rate. Abiraterone acetate was found to dramatically increase the overall survival compared to placebo (14.8 versus 10.9 months, *P* < 0.001). This benefit was observed across all patients regardless of performance status, sites of metastatic disease, and number of prior chemotherapy regimens received. In addition, abiraterone acetate was found to be superior over placebo for all secondary endpoints, including time to PSA progression (10.2 versus 6.6 months, *P* < 0.001), progression-free survival (5.6 versus 3.6 months, *P* < 0.001), and PSA response rate (29% versus 6%, *P* < 0.001). The drug was well tolerated with fluid retention and hypokalemia being the most common side effects. Based on these compelling clinical results, abiraterone acetate and prednisone were approved by the FDA in 2011 to treat docetaxel-refractory CRPC patients. 

The role of abiraterone acetate in a pre-chemotherapy setting is currently being explored in the COU-AA-302 Phase III clinical trial (NCT887198). Preliminary data from this trial of 1088 CRPC patients who have not been pretreated with docetaxel demonstrated that abiraterone acetate shows a trend in improving OS, progression-free survival, and time to chemotherapy initiation [35]. 

In addition to abiraterone, the nonsteroidal CYP17 inhibitor TAK-700 is currently being investigated in Phase III clinical trials composed of either docetaxel-refractory and chemotherapeutic-naïve CRPC patients. While these two compounds have a similar mechanism of action, one notable difference is that TAK-700 is a reversible inhibitor [36, 37]. In a Phase I/II trial, where patients received TAK-700 and prednisone, 41–63% of patients demonstrated a ≥50% decrease in PSA response rates at 12 weeks [38]. The most common adverse events were fatigue (72%), nausea (44%), and constipation (31%). It will be interesting to see if TAK-700 will be efficacious in a post-abiraterone setting (and vice versa). 

## 4. Sipuleucel-T

Sipuleucel-T was the first cellular immunotherapeutic to be approved by the FDA to treat cancer. Quite different than other drugs against CRPC, this treatment first isolates autologous peripheral blood mononuclear cells (PBMCs) from each patient via leukapheresis and then primes the isolated cells with the recombinant fusion protein prostatic acid phosphatase—GMCSF. This causes the activation and expansion of the autologous antigen-presenting cells (APCs), lymphocytes, and other cells [39, 40]. While questions still remain about the mechanism of action, it is believed that APC lead to the activation, recruitments, and subsequent destruction of cancerous cells expressing prostatic acid phosphatase.

The clinical development of sipuleucel-T has been controversial. In the first Phase III trial (NCT5947), 127 patients with asymptomatic metastatic CRPC were randomized 2 : 1 with one group receiving sipuleucel-T primed PBMCs and the other receiving PBMCs that were not treated [41]. The primary endpoint of the study was time to disease progression, while all patients were followed for survival. Following completion of the study, it was found that sipuleucel-T did not meet its primary endpoint. However, the median survival was 25.9 months for patients who received sipuleucel-T compared with 21.4 months in patients receiving control treatment (*P* = 0.01). To expand the population size, a second small Phase III trial (NCT1133704) was conducted with 98 asymptomatic metastatic CRPC patients [41]. When the results from this trial were combined with those of the earlier D9901 trial, sipuleucel-T showed a significant improvement in OS compared to placebo (23.2 versus 18.9 months, *P* = 0.01). Nevertheless, as OS was not the primary endpoint in either trial the FDA required further evidence in support of sipuleucel-T efficacy claim. Therefore, a larger third Phase III trial was initiated with 512 metastatic CRPC patients (IMPACT; NCT65442) [9]. The trial design was similar to the previous studies with one group receiving sipuleucel-T and the other receiving inactivated PBMCs. The median OS was 25.8 months for sipuleucel-T-treated patients compared with 21.7 months for patients receiving placebo with an adjusted HR for death of 0.78 (95% CI 0.61–0.98), representing a relative reduction in the risk of death of 22% (*P* = 0.03). In fact, further analysis of the IMPACT trial suggested that the OS benefit may be greater than previously published (4.1 months) [42]. Despite this survival benefit, both the PSA response and objective radiological response were not different in the two treatment arms. Adverse events from sipuleucel-T included chills, fatigue, fever, dyspnea, back pain, nausea, joint ache, headache, and local injection reaction. Most occurred within 1 day of infusion and were resolved within 24–48 hrs. Based on these clinical results, the FDA approved sipuleucel-T in 2010 for the treatment of asymptomatic or minimally symptomatic metastatic CRPC patients. Following approval and subsequent release of clinical data, there has been criticism of this trial, particularly with regard to age-related survival. Surprisingly, those patients <65 years old did not have any benefit (HR = 1.41) while those >65 years old demonstrated increased OS (HR = 0.58). While being unproven, it has been suggested that this age-related difference may be due to a difference in the number of PBMCs that were reinfused in the two treatment arms with the sipuleucel-T arm receiving a considerably larger number of leukocytes [43]. Whatever the cause, this age-related difference in efficacy is an important factor for the clinical utilization of sipuleucel-T. 

Despite being a remarkable scientific achievement, due to its cost, complicated treatment regimen, and the large number of newly approved competing therapeutics to treat CRPC, the utilization of sipuleucel-T has been lower than forecasted. Furthermore, the observed increase in OS in the absence of a change in progression-free survival or PSA response presents a challenge for clinicians. Using traditional markers of response to treatment in CRPC, there is no evidence that sipuleucel-T exerts a measurable antitumor activity. Sipuleucel-T's impact on the natural history of the disease is somewhat perplexing as there is no evidence of tumor burden reduction after treatment with this immune compound. Without these efficacy biomarkers, it will be challenging to incorporate sipuleucel-T in the treatment protocols.

## 5. Alpharadin

Over 80% of patients with CRPC have bone metastasis, which can cause bone pain, pathologic facture, pancytopenia, and spinal cord compression. These are, therefore, a major cause of both morbidity and mortality in late-stage prostate cancer patients. To reduce the number and severity of these skeletal-related events (SRE), the bisphosphonate zoledronic acid is being commonly used. 

Alpharadin is a bone targeting therapeutic comprised of the alpha-emitting isotope radium-223. This calcium mimetic naturally accumulates in the bone mineral hydroxyapatite, which is located at both in and around the metastatic deposit. Given the short path length of alpha emission, the radium-223 located in the hydroxyapatite will expose the metastatic tumor site to radiation, while limiting the damage to the soft tissue and bone marrow [44]. This elegant mechanism of action limits systemic adverse events that are observed with beta-emitting radiopharmaceuticals including strontium-89 and samarium-153 [34, 45]. 

In a Phase II trial of 64 CRPC patients scheduled to receive local-field external-beam radiation therapy to relieve pain from bone metastasis, alpharadin both prolonged the time to SRE and increased the OS [46]. Importantly, very few patients had grade 3-4 hematological toxicities, with the most common adverse events being nausea, bone pain, fatigue, diarrhea, vomiting, and constipation. Data from the ALSYMPCA (NCT699751), Phase III study of Alpharadin, has just recently been published [10]. In this trial, 921 CRPC patients with ≥2 symptomatic bone metastases and ineligible for or postprogression to docetaxel were randomized 2 : 1 and treated with either alpharadin and docetaxel or placebo and docetaxel. In the planned interim analysis (*n* = 809) alpharadin significantly improved OS compared to placebo (median 14.0 versus 11.2 months, *P* = 0.00185). In addition, the time to first SRE was lower in the alpharadin group (13.6 months) compared to placebo (8.4 months) (*P* = 0.00046). Based on these results, alpharadin has been granted Fast Track designation and is currently waiting for FDA approval. 

Further therapeutics have also been developed to reduce SRE in CRPC patients. Denosumab is a fully human monoclonal antibody directed against nuclear factor-kappa *β* ligand (RANKL), a key mediator of osteoclast formation, function, and survival. In metastatic prostate cancer, the invading cancer cells create an imbalance in the RANKL ratio that cause the bone structure to be weakened by osteoclast-mediated bone destruction [47]. In a Phase III study of 1904 patients with bone metastasis, denosumab was found to be superior to zoledronic acid in delaying the median time to first SRE (20.7 months versus 17.1 months, *P* = 0.002) [35]. The times to first and subsequent on-study SREs were significantly reduced by 18% in the denosumab arm. Overall disease progression and survival rates were similar. No major differences in adverse events were observed between the groups. A higher incidence of hypocalcemia was observed in those treated with denosumab (68 versus 56% of patients). Flu-like symptoms including back pain, nausea, fatigue, and anorexia were less compared to zoledronic acid (8.7 versus 20.2%, resp.). As significant adverse event observed in both the denosumab and zoledronic acid arms was osteonecrosis of the jaw (2% versus 1%, resp., *P* = 0.09). Most patients developing this complication had a history of tooth extraction, poor oral hygiene, or use of a dental appliance. Based on these results, FDA approved denosumab for preventing SREs in patients with bone metastases from solid tumors. Further to this indication, a Phase III trial was also conducted to test the effect of denosumab in delaying the formation of bone metastasis in nonmetastatic CRPC patients. In this study, over 1400 patients with nonmetastatic CRPC considered at high-risk for developing bone metastases (PSA ≥ 8 or PSA doubling-time ≤ 10 months or both) were randomized to receive either denosumab or placebo. Treatment with denosumab significantly increased both bone metastasis-free survival compared with placebo (29.5 versus 25.2 months, *P* = 0.028) and time to first bone metastasis (33.2 versus 29.5, *P* = 0.032). While denosumab was shown to delay the formation of bone metastasis in this study [48], the FDA Oncologic Drugs Advisory Committee voted against its clinical approval as the risks of the drug outweighed the potential benefits. 

## 6. Compounds Currently in Clinical Development

In addition to these newly approved therapeutics, there are several additional agents that show considerable promise to treat late-stage prostate cancer patients. 

OGX-011 is a second-generation phosphorothioate antisense oligodeoxynucleotide that is currently in late-stage clinical development. Targeting the clusterin protein, this therapeutic has demonstrated very good efficacy in preclinical models [49–51]. Importantly for antisense therapeutics, in clinical trials OGX-011 demonstrated reproducible pharmacokinetics with a relatively long *T*_1/2_ of 2-3 hrs and proportional increases in plasma concentrations and tissue concentration with increasing doses [52]. In a Phase II trial, chemotherapy-naïve CRPC patients were treated with docetaxel and randomized to receive either open-label OGX-011 or nothing [53]. Following a 35-month follow-up period, the PSA response rate was similar (58% with OGX-011 and docetaxel versus 54% docetaxel alone). In addition, the median progression-free survival did not differ significantly between the two arms. The median overall survival benefit was higher in the OGX-011 arm (23.8 months with OGX-011 and docetaxel versus 16.9 months docetaxel alone). In the second Phase II trial, the ability of OGX-011 to resensitize docetaxel-resistant CRPC to docetaxel was assessed. In this trial, 42 patients who were treated with OGX-011 and prednisone were randomized to either receive docetaxel or mitoxantrone. The median OS was longer in the docetaxel arm (15.8 months) compared to the mitoxantrone arm (11.5 months). In addition, many of the secondary endpoints including time to pain progression and PSA decline of >50% were considerably better in the docetaxel arm. This study demonstrated that OGX-011 and docetaxel may be advantageous in a patient who has been previously treated with docetaxel [54]. Accrual of >1000 CRPC patients have just recently been completed for the multicenter, Phase III trial (SYNERGY; NCT1188187), designed to assess whether the addition of OGX-011 to docetaxel-based chemotherapy can extend OS of men with metastatic CRPC. While the clinical development of antisense therapeutics have been challenging, the recent FDA approval of mipomersen suggests that this class of compounds may come to maturity [55]. 

Cabozantinib is a tyrosine kinase inhibitor that has been demonstrated to inhibit the RET, KIT, AXL, FLT3, MET, and VEGFR2 through which it causes a reduction of angiogenesis, cellular invasion, tumor growth, and metastasis [56]. It has demonstrated positive clinical results in a Phase II trial of 171 CRPC patients, with 72% of patients showing regression of soft-tissue lesions [57]. The most common grade 3 adverse events observed were fatigue (16%), hypertension (12%), and hand-foot syndrome (8%). Based on these results, recruitment is currently ongoing for a Phase III trial in post-enzalutamide/abiraterone patients (NCT1605227). 

## 7. Conclusions

With the introduction of these new therapeutics, the treatment of late-stage prostate cancer is dramatically changing. Yet, among the excitement of this rapid transformation many questions still remain. Specifically, for which patients, at what time, and in what sequence should these new treatments be utilized? These are not trivial questions and there is a considerable need for new guidelines to provide optimal treatment for CRPC patients. In addition, while these therapeutics have been approved in a post-docetaxel indication, the utilization of these new drugs before cytotoxic chemotherapy is very promising. Undoubtedly as the evidence becomes more solid, various treatment algorithms will be proposed by established clinical authorities and docetaxel-based chemotherapy will be used later in the course of the disease. For the time being, using these drugs in an off-label setting is based on individualized treatment decisions that are given after a thorough discussion about the possible benefits and drawbacks. Financial issues may be troublesome, especially for off-label use and in countries that cannot provide free public access to these drugs for the time being. Unfortunately, this off-label use will have a negative impact on the drug affordability and hence on the treatment compliance. Nevertheless, as these drugs obtain additional evidence-based support favoring their use in various stages of CRPC, patients will no longer be “destined” to use a cytotoxic drug. 

Despite the considerable success, we must remember that these new therapeutics only prolong median overall survival a few additional months. Given both the considerable cost of these new therapeutics and heterogeneity of late-stage prostate cancer there is an unmet need to identify those patients who will best respond to treatment. With the explosion in sequencing throughput, the scientific community is quickly learning more about the molecular mechanisms of cancer. The intersection of this basic research with clinical practice represents a tremendous opportunity with many challenges. 

Fundamentally, these newly approved therapeutics represent a significant shift in the treatment paradigm for CRPC patients. As we begin to learn how to utilize these drugs more efficiently, the survival and quality of life for CRPC patients will continue to increase. 

## Figures and Tables

**Table 1:** Treatments that have demonstrated OS benefit in Phase III trials with CRPC patients.

Treatment	Trial name and NCT identifier	Patient size	Indication	Control arm	Median OS in months (drug versus control)
Cabazitaxel/prednisone	TROPIC (NCT417079) [6]	755	Post-docetaxel	Mitoxantrone and prednisone	15.1 versus 12.7
Abiraterone/prednisone	COU-AA-301 (NCT638690) [7]	1195	Post-docetaxel	Placebo and prednisone	14.8 versus 10.9
Enzalutamide	AFFIRM (NCT974311) [8]	1199	Post-docetaxel	Placebo	18.4 versus 13.6
Sipuleucel-T	IMPACT (NCT65442) [9]	512	Post-docetaxel	PBMCs control	25.8 versus 21.7
Alpharadin	ALSYMPCA (NCT699751) [10]	922	Bone metastasis	Placebo and best standard care	14.0 versus 11.2
